# Esports Physical Exercise/PerformanceMatrix 1.0 Country Factsheets: a protocol for national, regional, and global annual assessment

**DOI:** 10.3389/fpubh.2024.1497552

**Published:** 2024-11-21

**Authors:** Bo Long, Agudamu, Radenko M. Matic, Stevo Popovic, Te Bu

**Affiliations:** ^1^College of Physical Education, Hunan Normal University, Changsha, China; ^2^Graduate School of Social Welfare, Sungkyunkwan University, Seoul, Republic of Korea; ^3^Faculty of Sport and Physical Education, University of Novi Sad, Novi Sad, Serbia; ^4^Faculty for Sport and Physical Education, University of Montenegro, Niksic, Montenegro; ^5^Western Balkan Sport Innovation Lab, Podgorica, Montenegro; ^6^K-CLUB, Korea University, Seoul, Republic of Korea

**Keywords:** esports, exercise, performance, monitoring, surveillance, system

## Abstract

This protocol helps evaluators gather current data and prepare annual assessments based on specific indicators to provide insights into physical activity among esports players and identify the challenges they face. This manuscript aims to develop a methodology for creating a standardized monitoring system to assess physical exercise and performance in esports players at national, regional, and global levels. This study protocol proposes 20 online sociodemographic indicators to help characterize participating countries and outline each country’s demographic profile. Additionally, this protocol proposes nine content indicators specifically designed to assess physical exercise and performance in esports players. A separate analysis will be required to evaluate each content indicator using a 10-point grading scale. This study protocol will facilitate the annual meetings of national evaluators (researchers) to produce reports, thereby fostering effective and dynamic linkages between research and practice.

## Introduction

Esports is commonly defined as “an organized and competitive approach to playing computer games” (1); however, it is essential to note that “all esports are video games, but not all video games are esports.” While other video games are primarily designed for entertainment, esports prioritize competition, leading to its gradual recognition as a legitimate form of sports competition (2).

Esports is increasingly recognized as a competitive sport, with unprecedented growth that has transformed it into a widely acknowledged industry. Although esports events vary from small tournaments to competitions held among close friends, their popularity reflects the market’s potential and the global audience’s growing appetite for international games with million-dollar prize pools at stake. Notably, in 2022, the esports sector surpassed USD 1.45 billion in revenue and is projected to grow from USD 1.72 billion in 2023 to USD 6.75 billion by 2030, at an annual growth rate of over 21.15% during the forecast period (3).

Moreover, esports has emerged as a new phenomenon in scientific research (4), attracting new talents, serving as a prime target for investments, and playing a crucial participant at the intersection of contemporary technology, media, and culture. This environment has also brought about various social concerns within esports, including issues of gender equity, diversity, cheating, doping, and physical and mental health challenges. Researchers are particularly concerned about the sedentary lifestyle associated with esports and the long hours players spend sitting in front of a computer or console (5).

It is widely recognized that a predominantly sedentary lifestyle can negatively impact the health of both the general population and esports players (6). Reports indicate that daily physical inactivity among esports players ranges from 5.5 to as much as 11 h per day (7–9). Therefore, reducing the adverse effects of physical inactivity on the cognitive skills that are highly correlated with success in esports is essential.

While researchers are paying more attention to the well-being of esports players, the development of specific health promotion strategies is still in its early stages. However, the scientific community is increasingly emphasizing the need to incorporate organized physical exercise into the daily training routines of esports players, due to its broad physiological benefits and clear cognitive improvements.

Several national, regional, and global monitoring systems track physical activity in the general population. These systems compare different surveillance methods developed by various researchers to monitor physical activity behavior and support physical activity assessment. There is currently no system supporting physical activity/exercise aimed at enhancing the performance and health of esports players, even though many researchers conducted independent studies with similar objectives. For this reason, tracking specific indicators over time is a crucial surveillance tactic that enables the assessment of changes in esports players’ behavior. This manuscript aims to develop a methodology that will primarily establish a standardized monitoring system for determining physical exercise and performance in esports players at the national level (in each country) while also extending its application to regional (across different geographical regions) and global (worldwide) assessments. This protocol was developed based on the best practices established by the authors in similar cases. It will allow representatives from various countries to collect up-to-date data and produce a comprehensive annual assessment through several specific indicators. This approach ensures that the outcomes include the most relevant evidence regarding physical activity/exercise for esports players in each country, as well as identifying specific barriers to physical activity/exercise. Once the evaluation is completed in each country, the responses will be summarized for reporting and research. Simultaneously, the Country Factsheets, a resource aggregating statistics related to physical activity/exercise levels among esports players, will be developed and disseminated to comprehensively understand the global variation in esports players’ physical activity/exercise-related indicators and critical influences. More significantly, the Country Factsheets serve as an advocacy tool, holding decision-makers accountable and urging them to take action on how stakeholders may implement new initiatives, programs, and policies to promote healthy surroundings and increase esports players’ physical activity and health.

## Methods and analysis

A team of content experts from the regional project titled ‘Report and Content Analysis on Physical Exercise and Performance of Esports Players’ (conducted in Serbia and Montenegro) and its international collaborators from China and Korea has developed a study protocol to collect data and produce an annual assessment of the esports industry’s role in promoting and facilitating physical activity and exercise opportunities for esports players using the list of specific indicators (to which grades will be assigned).

This study protocol will initiate the national evaluators (researchers) to meet annually to review the most relevant evidence on physical activity/exercise for esports players from each country and produce national reports (the Country Factsheets) to ensure effective and dynamic linkages between research and practice. Therefore, the Country Factsheets will play a key role in informing discussions that will lead to action on the physical inactivity of esports players and tangible improvements in the health of esports players and their performance in the future. This study protocol was developed based on the authors’ previous experience with the leading methodologies for the physical activity assessment of the general population (10–12), and it will assist individuals and groups working to promote physical activity/exercise for esports players. This protocol, as described in this peer-reviewed article, is also published on Research Square: https://doi.org/10.21203/rs.3.rs-4066825/v1 and includes supporting documents (1–4) available for reference. The Country Factsheets will be the primary output of this study protocol that will be developed through a harmonized and transparent process by collecting and collating the best available evidence and always produced in both short and expanded versions in English and disseminated through media and public awareness activities, undertaking knowledge exchanges through scientific conferences and workshops, and developing key partnerships with all interested stakeholders.

This study protocol proposes 20 sociodemographic indicators (country, total population, urban population, human capital index, Gross Domestic Product (GDP) *per capita*; GDP growth; unemployment; literacy rate; government expenditure on education; government expenditure on recreational and sporting services; individuals using the internet; mobile cellular subscriptions; fixed broadband subscriptions; high-technology exports; public health expenditure; life expectancy at birth; physical activity prevalence; deaths due to physical inactivity; national physical activity plan; and esports national federation) that are available online and it will be compiled to identify the characteristics of the participating countries and describe country’s demographic profile (Table 1).

**Table 1:** Country’s demographic profile.

Questions
Country for which you are providing responses:
Total population (number of people):
Urban population (%):
Human capital index (number between 0 and 1):
GDP *per capita* (current US$):
GDP growth (annual %):
Unemployment, total (% of total labor force):
Literacy rate, adult total (% of people ages 15 and above):
Government expenditure on education, total (% of government expenditure):
Government expenditure on recreational and sporting services, total (% of government expenditure):
Individuals using the Internet (% of population):
Mobile cellular subscriptions (per 100 people):
Fixed broadband subscriptions (per 100 people):
High-technology exports (current US$):
Public health expenditure (% of GDP):
Life expectancy at birth, total (years):
Physical activity prevalence (%):
Deaths due to physical inactivity (%):
National physical activity plan (Y/N):
Esports national federation (Y/N):

On the other hand, this protocol proposed nine content indicators specifically designed to assess physical exercise and performance in esports players (overall physical activity/exercise; formal physical activity/exercise; informal physical activity/exercise; active transportation; sedentary behaviors; physical performance; esports organization; community and environment; and government) as described in Table 2.

**Table 2:** Esports PEP Matrix 1.0 indicators and benchmarks used to guide the grade assignment process.

Indicator	Benchmark
Overall physical activity/exercise	% of esports players who meet the Canadian Sedentary Behaviour Guidelines (15), which recommend that aged group 18–64 years old accumulate at least 150 min of moderate to vigorous aerobic physical activity throughout the week; muscle strengthening activities using major muscle groups at least twice a week; and several hours of light physical activity, including standing.
Formal physical activity/exercise	% of esports players who participate in organized (formal) physical activity/exercise programs within esports organization.
Informal physical activity/exercise	% of esports players who participate in unorganized (informal) physical activity/exercise at any intensity for more than 2 h a day. % of esports players who report being outdoors for more than 2 h a day, without sitting or lying with low energy expenditure.
Active transportation	% of esports players who use active transportation, such as wheeling, walking and cycling, to get to and from places they visit on the daily bases.
Sedentary behaviors	% of esports players who meet the Canadian Sedentary Behaviour Guidelines (15), which recommend that aged group 18–64 years old spent at most 8 h a day sitting or lying with low energy expenditure, while awake, in the context of occupational, educational, home and community settings, and transportation.
Physical performance	Average percentile values achieved on certain physical fitness indicators based on the normative published by Hoffmann et al. (16).
Esports organization	% of esports organization with active institutional policies such as daily physical activity/exercise programs. % of esports organizations where the majority of esports players are taught by a certified physical activity/exercise specialists. % of esports organizations where the majority of esports players have regular access to facilities and equipment that support physical activity/exercise.
Community and environment	% of esports players who report having physical activity/exercise facilities or programs available to them in their community and environment (e.g., home, neighborhood, school, work, *et cetera*).
Government	% of esports organization that report receiving any funds and resources for the implementation of physical activity/exercise programs for esports players. % of esports organization that report any evidence of leadership in providing any specific type of physical activity/exercise opportunities for esports players. % of esports organization that report any demonstrated progress through the key stages of public policy making.

The authors of this study protocol will regularly collect input from their stakeholders to improve and refine the list of indicators each year and request external evaluation that will consider its inputs, outputs, and immediate, intermediate, and long-term outcomes.

To evaluate each of the content indicators, a separate analysis has to be conducted, and a 10-point grading scale will be employed (10 = exceptional; 9 = excellent; 8 = very good; 7 = good; 6 = fairly good; 5 = satisfactory; 4 = quite satisfactory; 3 = poor; 2 = very poor; 1 = failing; 0 = without reliable information). The grades will be awarded based on data found in available scientific articles in the last 10 years and secondary data sources such as governmental and nongovernmental reports and online content from a specific period. Then, the findings will be synthesized, and the grade assessment process will be completed using the grading framework described in Table 3. Each indicator will be discussed until a grade consensus is reached among the evaluators.

**Table 3:** Esports Physical Exercise/Performance Matrix 1.0 grading system.

Grade	CI	Description
10	90% ≤	Exceptional
9	80–89%	Excellent
8	70–79%	Very good
7	60–69%	Good
6	50–59%	Fairly good
5	40–49%	Satisfactory
4	30–39%	Quite satisfactory
3	20–29%	Poor
2	10–19%	Very poor
1	≤10%	Failing
0	Insufficient or inadequate information to assign a grade	Incomplete

CI – Class intervals represent the difference between the upper class limit and the lower class limits of available information.

The reliability of content analysis in this study will involve operationalizing the concept of physical exercise and performance in esports players, training coders to implement this concept, and evaluating coder reliability in applying these definitions (13). The interjudge agreement index will be calculated using Cohen’s kappa coefficient (*κ*), as suggested by McHugh (14), to address potential bias issues. The electronic databases (SportDiscus, Scopus, PubMed/MEDLINE, and Web of Science) will be used to search research articles, Open Access Theses and Dissertations (OATD), and Networked Digital Library of Theses and Dissertations (NDLTD) to search theses and dissertations, and Google to search other documents and online content needed to evaluate the content indicators. The full search syntax used for each database is described in Table 4.

**Table 4:** Full search syntax used for each database.

Database	Search syntax
Scopus	title-abs-key(“physical activity” or “physical excercise” or sedentar* or sitting) and title-abs-key(esport) and title-abs-key(country)
PubMed/MEDLINE	(“physical activity”[tw] OR “physical exercise”[tw] OR sedentar*[tw] OR sitting[tw]) AND (esport[tw] AND country[tw])
Web of Science, SportDiscus (through EBSCOhost) Open Access Theses and Dissertations (OATD), Networked Digital Library of Theses and Dissertations (NDLTD)	(“physical activity” or “physical exercise” or sedentar* or sitting) and (esport) and (country)
Google	“physical activity” or “physical exercise” or sedentar* or sitting and esport* and country

Replace the word “country” with the specific name of the country being investigated.

## Discussion and dissemination

This protocol empowers evaluators to collect standardized data and produce a comprehensive annual assessment. Through specific indicators, it seeks to provide robust evidence on esports players’ physical activity and identify the challenges they face.

The Country Factsheets primarily target esports organizations, policymakers, and nonprofit organizations involved in promoting health and physical activity among esports players. The role of the media in promoting the Country Factsheets is crucial, as it helps to raise awareness and encourage esports organizations to foster more physical activity among players. While the general public is not the primary audience, the Country Factsheets will receive significant media attention, reinforcing the importance of physical health in esports.

The dissemination strategy includes annual campaigns and targeted social media outreach to promote the Country Factsheets. These campaigns will focus on actionable recommendations for enhancing esports players’ physical activity and overall wellbeing.

Expected outcomes are divided into short-term, intermediate, and long-term goals. In the short term, the Country Factsheets will raise awareness among decision-makers and media outlets about esports players’ physical activity needs. Intermediate outcomes include policy and program development by government and non-government stakeholders to support esports players’ physical activity. In the long term, the study’s success will be reflected in increased rates of physical activity among esports players, contributing to their health and performance benefits over time.

Regarding this protocol’s limitations, it is essential to note that the methodology relies on publicly available data, which may vary in quality and comprehensiveness across countries, potentially affecting cross-country comparability. The 10-point grading scale used for evaluating indicators introduces some subjectivity. Although efforts are made to reach a consensus among evaluators and calculate interjudge agreement, individual interpretations of the data may still influence the grading process.

While the protocol assumes a standardized approach to assessing physical activity and esports performance, it may not fully account for cultural variations in these practices across areas. Additionally, access to esports-related infrastructure and technology varies, particularly in lower-income countries, which may affect the accurate representation of esports players’ physical activity and health behaviors.

To mitigate data inconsistencies across countries, the study will employ standardized indicators, provide annual training for evaluators, and use multi-source verification methods to ensure data reliability and comparability. For cases of missing or incomplete data, gap-filling techniques such as regional averages will be used, with any estimates transparently flagged. Data quality will also be ensured through external review by international experts and consensus-building guided by interjudge agreement metrics, such as Cohen’s Kappa.

This protocol establishes a comprehensive framework for assessing physical activity among esports players at national, regional, and global levels. The development of the Esports Physical Exercise/Performance Matrix and Country Factsheets provides a standardized approach for evaluating and promoting physical activity in the esports industry. This study’s unique focus on the health and performance of esports players addresses a significant gap in current research, laying the groundwork for evidence-based policies and interventions to improve player wellbeing and performance.
