# Corrigendum: Esports Physical Exercise/Performance Matrix 1.0 Country Factsheets: a protocol for national, regional, and global annual assessment

**DOI:** 10.3389/fpubh.2024.1547217

**Published:** 2025-01-17

**Authors:** Bo Long, Agudamu, Radenko M. Matic, Stevo Popovic, Te Bu

**Affiliations:** ^1^College of Physical Education, Hunan Normal University, Changsha, China; ^2^Graduate School of Social Welfare, Sungkyunkwan University, Seoul, Republic of Korea; ^3^Faculty of Sport and Physical Education, University of Novi Sad, Novi Sad, Serbia; ^4^Faculty for Sport and Physical Education, University of Montenegro, Niksic, Montenegro; ^5^Western Balkan Sport Innovation Lab, Podgorica, Montenegro; ^6^K-CLUB, Korea University, Seoul, Republic of Korea

**Keywords:** esports, exercise, performance, monitoring, surveillance, system

In the published article, there was an error in the article title. Instead of “Esports Physical Exercise/Performance Matrix 1.0 Country Factsheets: a protocol for national, regional, and global annual assessment study protocol,” it should be “Esports Physical Exercise/Performance Matrix 1.0 Country Factsheets: a protocol for national, regional, and global annual assessment.”

In the published article, there was an error in the legend for “Table 4. Full search syntax used for each database” as published. We have missed to add legend. The corrected legend appears below:

“Replace the word “country” with the specific name of the country being investigated.”

In the published article, there was an error in “Table 4. Full search syntax used for each database” as published. We would like to type “country” instead of “Serbia.” The corrected “Table 4. Full search syntax used for each database” and its caption appear below.

**Table 4:** Full search syntax used for each database.

**Database**	**Search syntax**
Scopus	title-abs-key(“physical activity” or “physical excercise” or sedentar^*^ or sitting) and title-abs-key(esport) and title-abs-key(country)
PubMed/MEDLINE	(“physical activity”[tw] OR “physical exercise”[tw] OR sedentar^*^[tw] OR sitting[tw]) AND (esport[tw] AND country[tw])
Web of Science, SportDiscus (through EBSCOhost) Open Access Theses and Dissertations (OATD), Networked Digital Library of Theses and Dissertations (NDLTD)	(“physical activity” or “physical exercise” or sedentar^*^ or sitting) and (esport) and (country)
Google	“physical activity” or “physical exercise” or sedentar^*^ or sitting and esport^*^ and country

Replace the word “country” with the specific name of the country being investigated.

The authors apologize for these errors and state that this does not change the scientific conclusions of the article in any way. The original article has been updated.

